# Contact between European bison and cattle from the cattle breeders’ perspective, in the light of the risk of pathogen transmission

**DOI:** 10.1371/journal.pone.0285245

**Published:** 2023-05-03

**Authors:** Daniel Klich, Anna Didkowska, Anna M. Pyziel-Serafin, Magdalena Perlińska-Teresiak, Aleksandra Wołoszyn-Gałęza, Krzysztof Żoch, Marek Balcerak, Wanda Olech

**Affiliations:** 1 Department of Animal Genetic and Conservation, Institute of Animal Sciences, Warsaw University of Life Sciences–SGGW, Warsaw, Poland; 2 Department of Food Hygiene and Public Health Protection, Institute of Veterinary Medicine, Warsaw University of Life Sciences (SGGW), Warsaw, Poland; 3 Museum and Institute of Zoology, Polish Academy of Sciences, Ustrzyki Dolne, Poland; 4 Borki Forest District, Kruklanki, Poland; 5 Department of Animal Breeding, Warsaw University of Life Sciences-SGGW, Warsaw, Poland; Universitat Autonoma de Barcelona, SPAIN

## Abstract

Pathogens transmitted between wildlife and domestic animals can pose a threat to endangered species, undermine conservation efforts in wildlife, and affect productivity and parasite control in domestic animals. There are several examples of pathogen transmission between European bison and other animals. The present study surveyed breeders from the vicinity of four large wisent populations in eastern Poland about observed contacts between wisent and cattle. Such contacts were noted by 37% of breeders, indicating a significant risk of contact between European bison and cattle in the study areas, even in the areas where the European bison live mainly in a forest complex, i.e., in the Borecka Forest. A higher potential risk of contacts between European bison and cattle was noted in the Białowieska Forest and the Bieszczady Mountains than in the Borecka and Knyszyńska Forests. In the Białowieska Forest, the risk of viral pathogen transmission resulting from contacts is higher (more direct contacts), and in the case of the Bieszczady Mountains, the probability of parasitic diseases is higher. The chance of contacts between European bison and cattle depended on the distance of cattle pastures from human settlements. Moreover, such contact was possible throughout the year, not only in spring and fall. It appears possible to minimize the risk of contacts between wisent and cattle by changing management practices for both species, such as keeping grazing areas as close as possible to settlements, and reducing the time cattle graze on pastures. However, the risk of contact is much greater if European bison populations are large and are dispersed beyond forest complexes.

## Introduction

Following its return from extinction, the European bison population has continued to grow, and its recent dynamic increase in population [1] has resulted in its status being changed to NT (near threatened) [2]. However, the current situation of the species continues to require active conservation measures due to the emergence of new threats [3,4].

This rapid growth in population size has been achieved through conservation efforts, but it results in high population densities within residential areas (usually forest complexes), which can lead to easier transmission of pathogens among individuals [5]. In the past, transmission of tuberculosis and currently thelaziasis led to a significant reduction in the number of wisents in southeastern Poland [6,7]. Thelaziasis is an eye disease of wild and domestic animals that eventually results in blindness. It is distributed worldwide by spirurid eyeworms of the genus *Thelazia*, with non-biting secretophagous flies of the genus *Musca* playing the role of intermediate hosts. The symptoms derive from the mechanical irritation of the conjunctiva and cornea, and toxic effects of parasitic metabolites [8,9]. Under pressure from their increasing population density, wisents are increasingly moving beyond the area of forest complexes [10,11]. This is associated with greater foraging on crops, which may cause negative health effects due to pesticides, or deficiencies in certain elements [12,13]. In addition, such foraging on agricultural land is also associated with a higher probability of contact with cattle.

Diseases common to wildlife and domestic animals can pose a threat to endangered species, undermine conservation efforts in wildlife, or interfere with productivity and parasite control in domestic animals. In addition, wildlife is the most likely source of emerging diseases for both humans and animals [14], such as the transmission of SARS-CoV-2 from wildlife to humans and domestic animals [15]. The phenomenon of pathogens being transmitted via wildlife-livestock contacts has been already widely described. For example, in the United States, it was estimated that about 80% of reported livestock diseases have a potential wildlife component [16]. Direct contact often requires temporal and spatial co-location between hosts, while indirect contact requires co-location with a certain time window [17]. When livestock and animal species are susceptible to the same pathogen, controlling disease in one can have an impact on the prevalence in the other. One good example being efforts to control brucellosis in livestock resulted in a lower prevalence in red deer (*Cervus elaphus*) [18]. Interactions with cattle also contribute to the occurrence of brucellosis in elk (*Cervus canadensis*) [19]. Similar associations have been identified for other diseases, such as tuberculosis in the case of free-living African buffalo (*Syncerus caffer*), American bison (*Bison bison*), red deer (*Cervus elaphus*), roe deer (*Capreolus capreolus*), wild boar (*Sus scrofa*) [20–23]. Presence of common wild ungulates on ranches were also noted in African countries [24]. In particular, eland (*Taurotragus oryx*), impala (*Aepyceros melampus*) and waterbuck (*Kobus defassa*) have been associated with a higher risk of wildlife-livestock pathogen transmission [25–27].

Bacterial diseases that occur in cattle may pose a potential threat to European bison in the case of direct contact, but also due to regular grazing on the same pastures. One of the greatest such threats is presented by tuberculosis, partly due to the relatively high environmental resistance of mycobacteria [28,29]. Animal transmission of tuberculosis remains an emerging problem [30,31]. Recent studies have also demonstrated the occurrence of antibodies to pathogens such as Schmallenberg virus (SBV), bluetongue virus (BTV), bovine viral diarrhea virus (BVDV), bovine herpesvirus type 1 (BoHV-1), *Mycoplasma* spp, *Brucella* spp, bovine adenovirus type 3 (BAdV-3), bovine parainfluenza type 3 (PIV-3), bovine respiratory syncytial virus (BRSV), *Toxoplasma gondii*, *Leptospira* spp. [32–34]. It has been suggested that contact with some of these pathogens may have resulted from contact with cattle [34]. According to Kwiecień et al. [35], cattle and European bison sharing common pastures are subject to a higher risk of *Trueperella pyogenes* transmission. European bison and cattle also share a variety of endoparasites from different classes [36], such as 11 species of protozoan parasites belonging to the genus *Eimeria* [37,38], as well as *Neospora caninum*, *Toxoplasma gondii*, and *Sarcocystis cruzi* [39–42]. Many parasitic nematode species are generalists that can infect multiple host species [43]. Thanks to the direct life cycle of gastrointestinal nematodes, it is possible for wild and domestic animals sharing the same grazing areas to ingest parasite larvae laid by different species [43]. Both European bison and cattle share the following abomasal nematodes, among others: *Ostertagia ostertagi*, *O*. *lyrata*, and the bloodsucking *Haemonchus contortus* [44,45]. Another parasite, *Ashworthius sidemi*, is an alien species that was first introduced to Polish wildlife with migrating Asian cervids, and then transferred to the Czech Republic with European bison translocated from Poland [46,47]. Pasture sharing can also lead to transmission of lungworm larvae (*Dictyocaulus viviparus*) between animals; although the parasite demonstrates some genetic variability, it belongs to a single species [37,48–50]. In addition, eyeworm infestations by the spirurid nematodes *Thelazia gulosa* and *T*. *skrjabini*, are frequently diagnosed in cattle, whereas their distribution to cervids is very limited [6,9].

Hence, it seems important to understand the possible contacts between European bison and cattle which can lead to the transmission of pathogens. Such knowledge would be valuable for protecting European bison and ensuring proper livestock management. Unfortunately, very little information on this topic is currently available in the literature. Many wildlife-borne diseases are known to affect the agricultural economy [e.g. 51] and limiting wildlife-livestock contacts is an important element of disease control strategy [52]. Therefore, it is important to identify mechanisms to minimize potential contacts between European bison and cattle so that they can be used in the event of a threat.

With this in mind, the present study surveys cattle breeders from the vicinity of four large populations of European bison in eastern Poland: the Borecka, Białowieska and Knyszyńska forests and the Bieszczady Mountains. The study was based on four hypotheses: 1) European bison have contact with cattle in all studied populations, but less so in Borecka Forest, where the population roams within the forest complex as a result of management methods. 2) Contacts between European bison and cattle are less frequent when cattle are kept near settlements. 3) Contacts between European bison and cattle occur throughout a few months of the year (spring and autumn). 4) Cattle breeders perceive the contacts between European bison and cattle as a serious threat to them and the cattle.

## Methods

**Fig 1:**
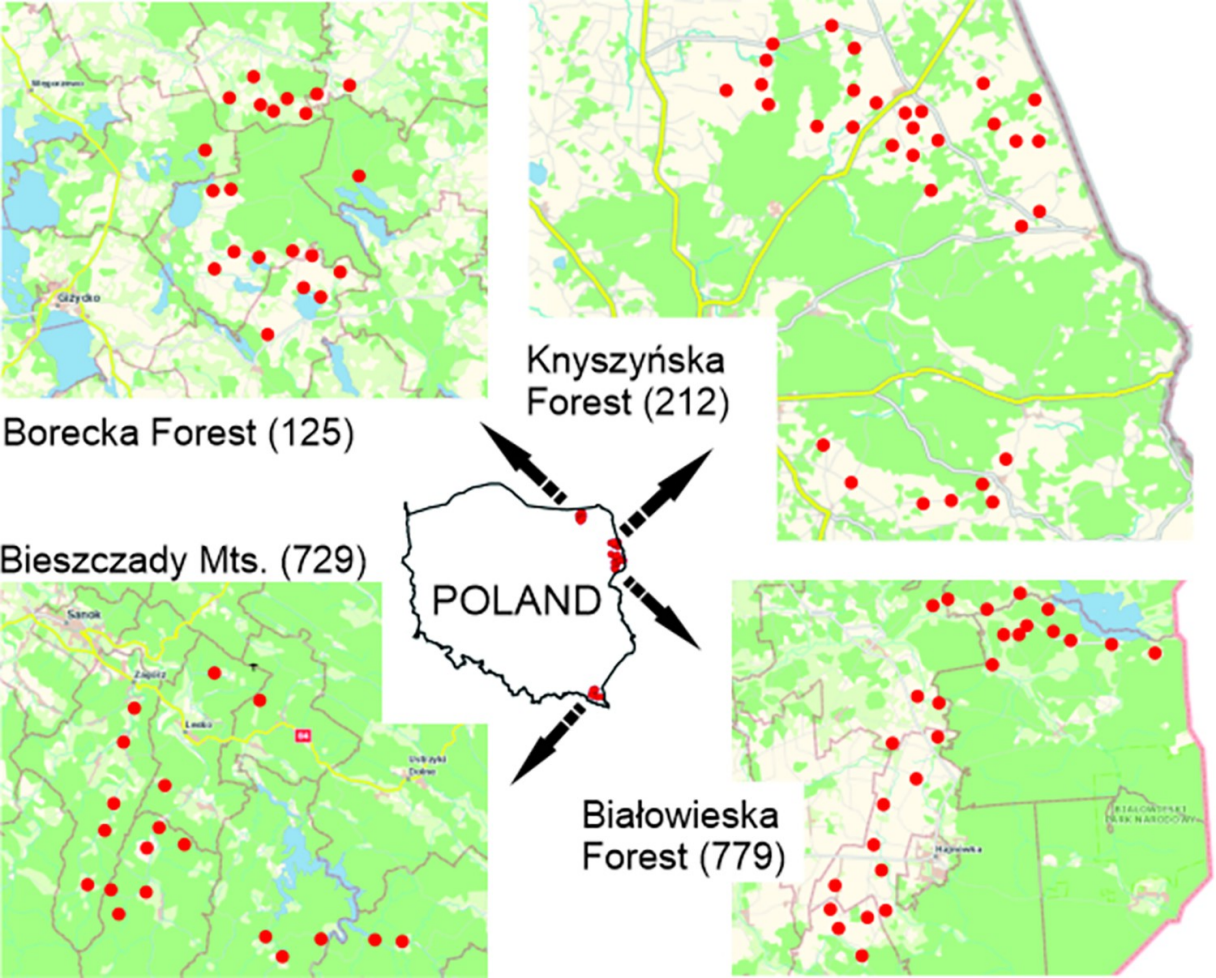
Locations of the villages where the surveys were conducted in given study area (with the number of European bison in brackets [53]). Background map was obtained from Geoportal.gov.pl.

The study was conducted near the four largest populations of European bison in eastern Poland: Białowieska, Borecka and Knyszyńska Forests in northeastern Poland and the Bieszczady Mountains in southeastern Poland (Fig 1). The Białowieska Forest covers an area of about 600 km^2^ (52°44’N, 23°47’E), and the European bison was first reintroduced there in 1952. The current population size (including adjacent areas) is estimated at 779 individuals [53]. For many years, European bison have used the areas outside the forest complex, i.e. agricultural land [10], which resulted in severe damage to crops [54]. The Borecka Forest is a dense forest covering an area of about 200 km^2^ (54°8’N, 22°8’E), isolated by agricultural land and lakes. The European bison was reintroduced in 1962 and the current population size is estimated at 125 individuals [53]. The population is actively managed and there is no significant agricultural damage [54]. The Knyszyńska Forest covers an area of about 1000 km^2^ (53°12’N, 23°28’E) and the European bison was reintroduced in 1974. Currently, the population of this species is estimated at 212 individuals [53]. European bison use the areas outside the forest complex in large numbers and cause considerable damage to agricultural crops [11]. The Bieszczady Mountains cover an area of more than 1,100 km^2^ (49°15’N, 22°25’E), but only part of this area is inhabited by the European bison, which were reintroduced in 1963. Unlike the rest of the study area, this area is mountainous and has undergone a significant change from cropland to predominantly woodland [55]. European bison mainly use forest habitats in this area, where they cause significant damage [56,57]. In addition, European bison also frequently cause damage to farms [58]. The current population size is estimated at 729 individuals [53].

Questionnaires (direct interviews) were administered to cattle breeders in the four study areas. A total of 208 questionnaires were completed, at least 50 in each study area: 54 in Białowieska Forest, 51 in Borecka Forest, 52 in Knyszyńska Forest, and 51 in Bieszczady Mountains. Respondents were selected using the snowball sampling method, which is suitable for studying hard-to-reach populations [59]. All respondents were informed of the purpose of the survey and principles of data collection, and consciously gave their verbal consent to conduct the survey. Consent was expressed in the presence of a witness, someone from the respondent’s family or another third party. If there was no witness, the interviewer certified in writing that oral consent had been obtained from the respondent. Surveys were not linked to respondents’ addresses and were anonymous, so no sensitive data were collected. Respondents were not restricted to age (except from being adult) or gender, and the only selection criterion for respondents was that they were owners (or managers) of cattle raised on pasture.

To test the hypotheses, nine questions were prepared for the breeders. The survey was divided into three sections:

- basic information (questions 1–5): location of the breeder’s home (village), number of cows on the pasture, breed, outdoor grazing time, distance of the pasture from settlements and the nearest forest, method of securing the cattle (e.g. electric fence),
- essential information on contacts of European bison with cattle (questions 6–7): whether the breeder observed such contact and how domestic animals reacted to it,
- risk assessment by the breeder (questions 8–9): questions on the threat of European bison to cattle and humans.

For question 6, "Have you or anyone in your family or your associates observed contact between European bison and cattle?", respondents had a choice of five response categories: 1) No, never (NO CONTACT), 2) European bison were seen in the same pastures as cattle but at different times (SHARING PASTURES), 3) European bison were seen at a distance from cattle (at least 100 m) (AT DISTANCE), 4) European bison were seen in close proximity but separated by a barrier (e.g., a fence) (SEPARATED), 5) European bison were seen in close proximity to cattle without barriers (less than 100m or between animals including mixed herds of cattle and European bison (NEAR). For categories 2 to 5, the respondent was also asked how many times a year and in what time period (in months) each potential contact occurred. If the respondent selected categories 3 to 5 in question 6, conditional question 7 was asked: “If contact was noticed, what type of reactions of cattle was observed?”.

Question 8, "Do you think direct contact with European bison poses a risk to cattle or humans?" allowed respondents to choose from five response categories: 1) Definitely not, 2) Rather not, 3) Hard to say, 4) Rather yes, 5) Definitely yes. If the respondent selected categories 4 or 5 in question 8, the conditional open-ended question 9 was asked, "Please indicate the type of risk to livestock and/or humans."

If the breeders declared more than one pasture, they were asked to indicate the most important pasture where the cattle graze most often and where the herd can be observed. In this way, each breeder was limited to one answer. The overall response rate was 92%, with the rates in each individual study areas being 93% in Białowieska Forest, 96% in Bieszczady Monuntains, 93% in Borecka Forest and 87% in Knyszyńska Forest. The lack of response to the questionnaires resulted from the inability to contact the breeder (not at home), or that the cattle were kept in closed barns rather than on pasture (in the Knyszyńska forest). None of the breeders refused to answer the questions. The main data obtained in this study is presented in S1 Table.

All data used in the analysis were taken directly from the survey data, including the distance of the pasture to the settlements, which was estimated by the breeders. The survey data were analyzed using a generalized linear model with a logistic link function and a binary response variable. In this model, the dependent variable was the observation of potential contact of European bison with cattle (SHARING PASTURES, AT DISTANCE, SEPARATED, and NEAR, labeled as 1) or the absence of such contact (NO CONTACT, labeled as 0). The explanatory variables in the model were: (A) distance of cattle grazing from human settlements (DIST _S), distance of cattle grazing from forest habitat (DIST _F), and study area (SITE). A correlation between DIST _S and DIST _F was checked prior to modeling. As the distance of cattle grazing from human settlements was statistically significant, another model was created with the same dependent variable and the distance from human settlements as the sole explanatory variable.

The risk perception was also checked for differences among breeders in the study areas. The Kruskal-Wallis test was used because the dependent variable was not normally distributed. The test used risk perception as a transformed variable (both cattle and human risk) on a five-point Likert scale, i.e., 1) Definitely not, 2) Rather not, 3) Hard to say, 4) Rather yes, 5) Definitely yes.

## Results

**Fig 2:**
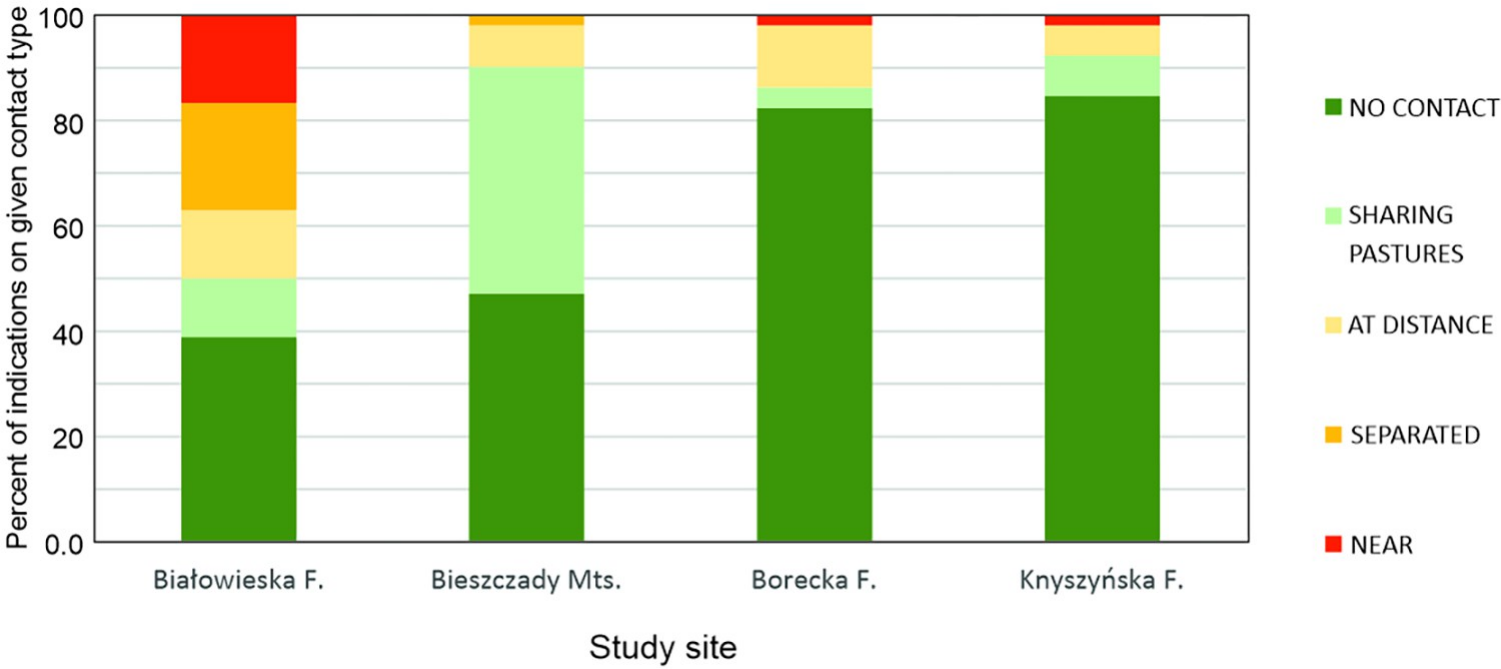
Percentage of forms of European bison contact with cattle at specific study sites, as reported by cattle breeders (see Methods for a detailed explanation).

In general, 37% (95%CI: 30.4–43.6%) of breeders reported potential contact between European bison and cattle. However, indirect potential contact (SHARING PASTURES) was most frequently reported among contact types (44.2% of all potential contact data; 95%CI: 33.1–55.3%). The presence of European bison at a considerable distance from cattle (AT DISTANCE) was also frequently indicated (26%; 95%CI: 16.2–35.8%). The other categories (SEPARATED and NEAR) together accounted for nearly 30% (95%CI: 19.6–40.0%) of all potential contacts. However, breeder observations varied across study areas. In the Białowieska Forest, breeders most frequently noticed potential contacts between European bison and cattle (more than 60%; 95%CI: 48.1–74.1%). Moreover, breeders most frequently noticed the presence of European bison among cattle (NEAR) comparing to other potential contact types (Fig 2). In the Bieszczady Mountains, more than 50% (95%CI: 39.2–66.6%) of breeders indicated potential contacts between European bison and cattle and SHARING PASTURES was most frequently noted among the potential contact types (43.1% of all potential contacts, 95%CI: 29.5–56.7%). In other study areas (Borecka and Knyszyńska Forests), potential contacts were observed less frequently (17.6; 95%CI: 7.1–28.1% and 15.4%; 95%CI: 5.6–25.2%, respectively) than in Białowieska and Knyszyńska forests (Fig 2).

**Fig 3:**
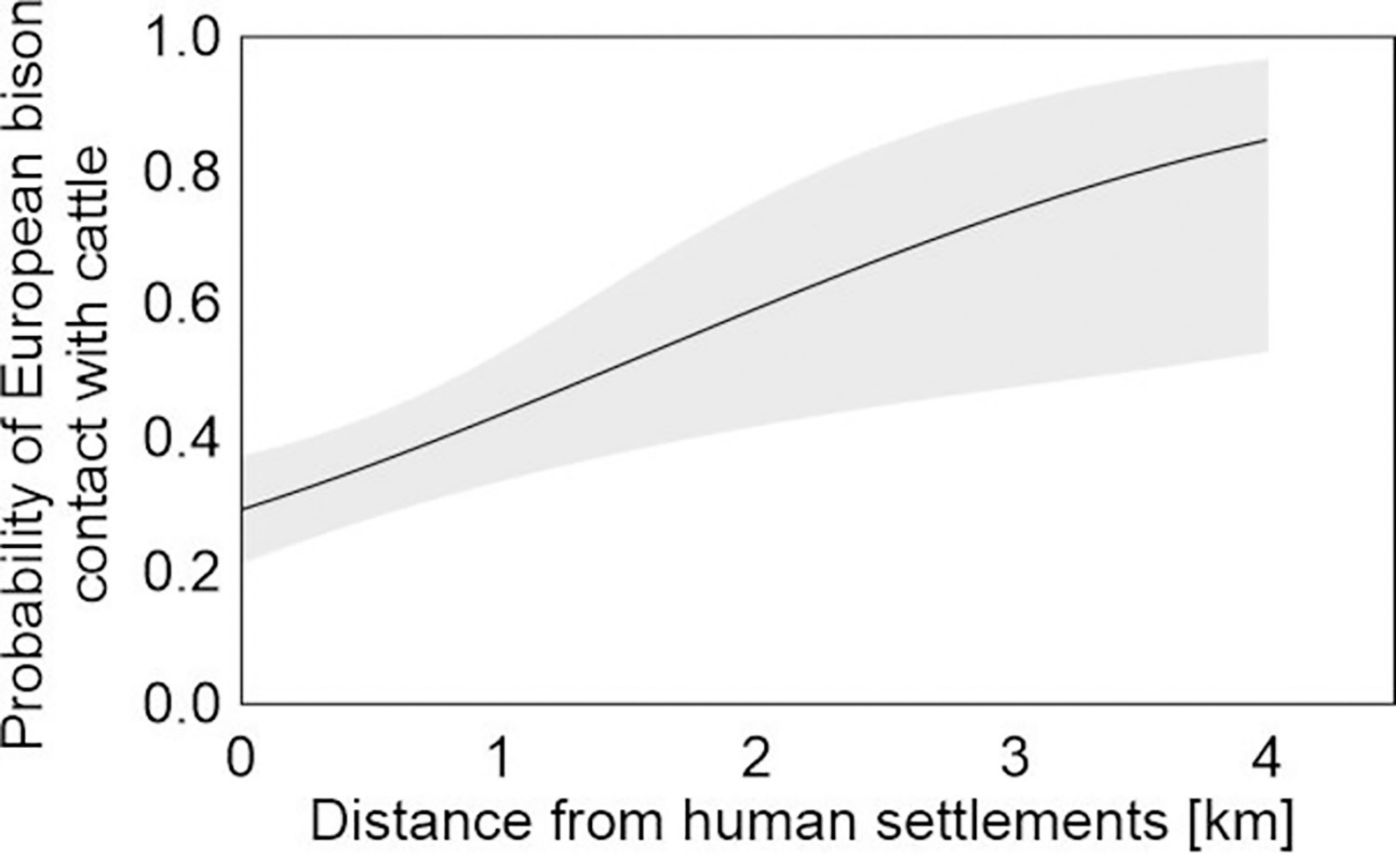
Probability (predicted mean and CIs—shaded area) of potential contact of European bison (direct and indirect) with cattle with distance of cattle pastures from human settlements (for the model: χ2 = 9.77, df = 1, p = 0.002).

The generalized linear binary model indicates that the distance of cattle pastures from human settlements and the study site had a significant influence on the potential contact between European bison and cattle (Table 1), with a greater distance being associated with a higher risk of potential contact. The distance between cattle grazing areas and human settlements could independently explain the risk of potential contact between European bison and cattle. The probability of potential contact was higher than 50% for pastures located 1.5 km from human settlements (Fig 3). However, this effect was not found in relation to the distance of cattle pastures from forest habitat. The frequency of potential contacts was higher in Białowieska Forest and Bieszczady Mountains than in Knyszyńska Forest, but the frequency of potential contacts did not differ between Borecka and Knyszyńska Forests (Table 1).

**Table 1:** Influence of distance of cattle grazing from human settlements (DIST _S) and forest habitats (DIST _F) and study site (SITE) on probability of potential contact of European bison with cattle in a generalized linear binary model; 0—reference category (overall model: χ2 = 44.36, df = 5, p < 0.001).

Source	B	SE	Lower CI	Upper CI	Wald χ^2^	p
Intercept	-1.691	0.4349	-2.544	-0.839	15.120	<0.001
DIST_S	0.539	0.2377	0.073	1.005	5.135	0.023
DIST_F	-0.081	0.1051	-0.287	0.125	0.599	0.439
SITE (Białowieska F.)	1.829	0.4916	0.866	2.793	13.847	<0.001
SITE (Bieszczady Mts.)	1.581	0.5080	0.585	2.577	9.686	0.002
SITE (Borecka F.)	-0.308	0.5796	-1.445	0.828	0.283	0.595
SITE (Knyszyńska F.)	0					

**Fig 4:**
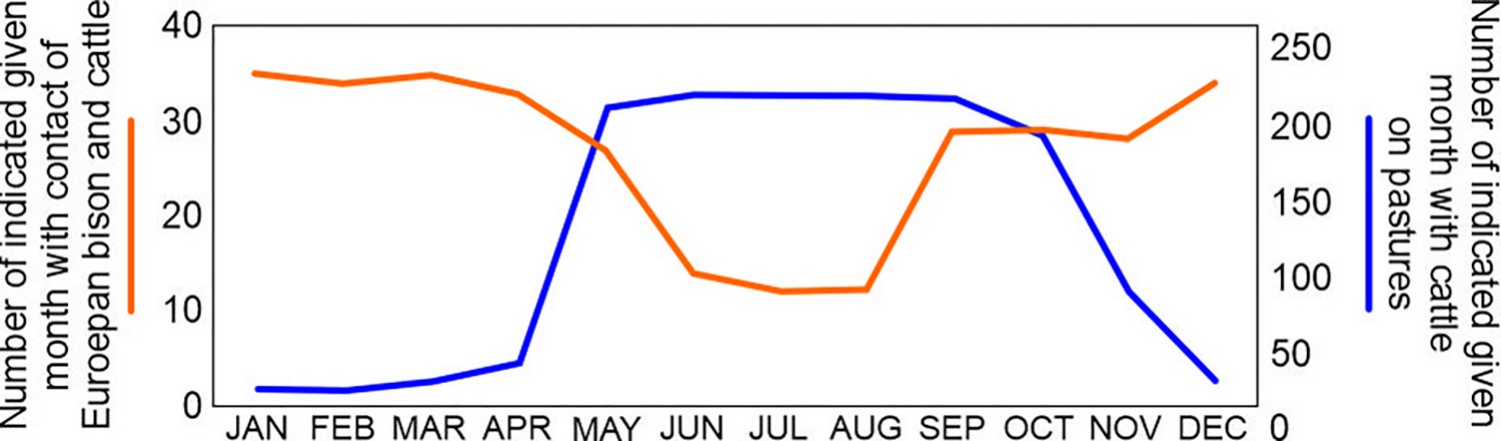
The overlap between the presence of cattle on pastures with their potential contact with European bison.

Cattle were mostly grazed from May to October, although some breeders reported keeping animals year-round (Fig 4). Breeders most frequently indicated that potential contact between European bison and cattle could occur from January through May and from September through December (Fig 4). The highest overlap of cattle on pasture and potential contacts is observed in May, September, and October (Fig 4).

**Fig 5:**
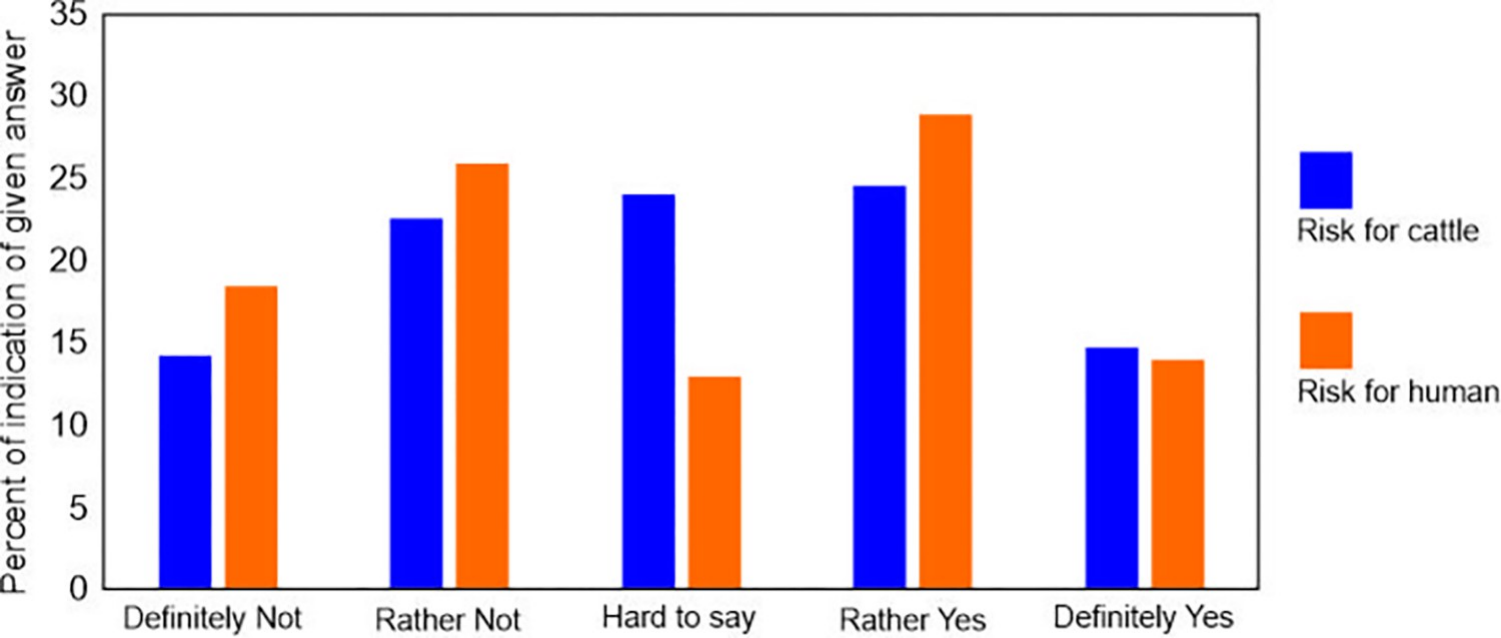
Percentage of responses to a question, "Do you think direct contact with European bison poses a risk to cattle or humans?".

**Fig 6:**
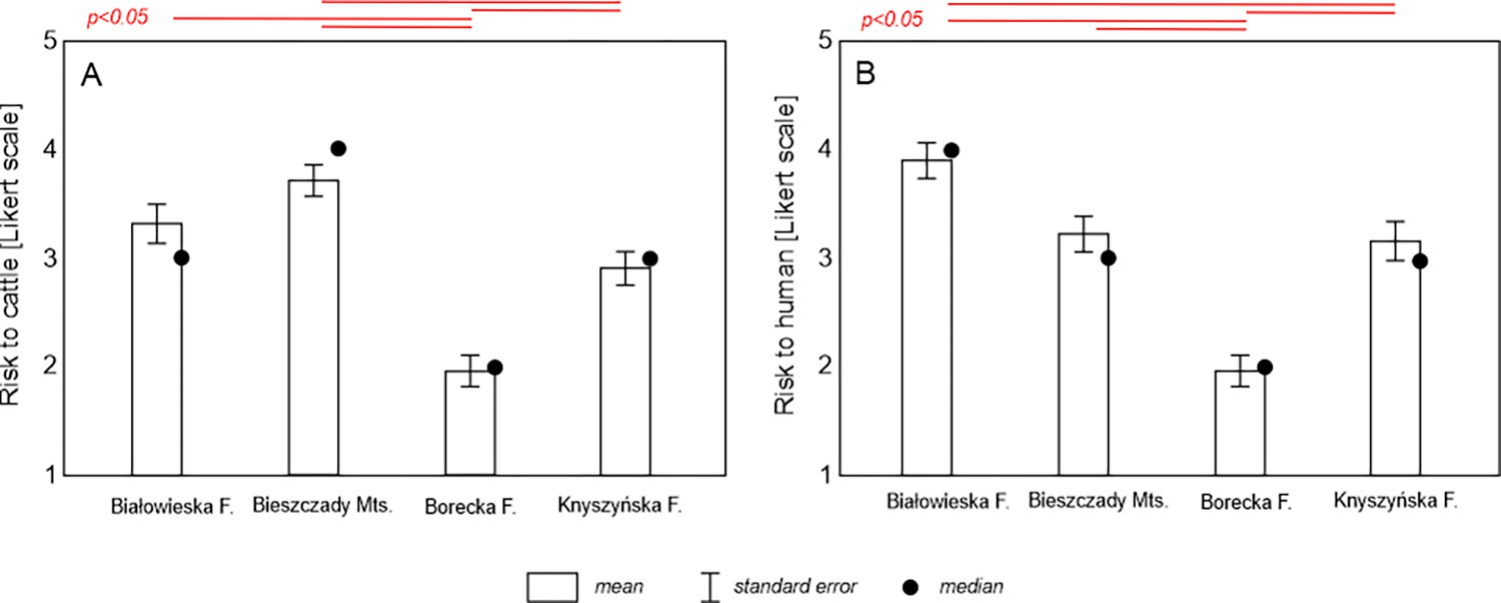
Cattle (A) and human (B) risk assessment of European bison and pairwise comparison using the Kruskal-Wallis test (red horizontal lines above bars indicate statistically significant differences).

Breeders rated the risk posed by European bison to cattle and humans as moderate (Fig 5). A similar percentage indicated no risk, although they indicated that the threat to cattle was lower than to humans (responses of "Rather yes" and "Definitely yes" accounted for 39.2% (95%CI: 32.5–45.9%) responses for cattle and 42.8% (95%CI: 36.0–49.6%) responses for humans. Among the threats to cattle, breeders primarily indicated the risk of pathogen transmission (43% of responses; 95%CI: 31.8–54.2%) and aggression from European bison (24.4% of responses; 95%CI: 14.7–34.1%). Threats also included scaring cattle, mating with cattle, destroying fences, and reducing the quality of pastures. With respect to humans, all of the breeders who responded to this question feared aggression from European bison. According to the breeders, the cattle usually reacted with fear or interest to the European bison. The lowest risk from the European bison to cattle and humans was reported in the Borecka Forest; respondents from this study area were demonstrated significantly different risk perception compared to all other sites (Fig 6). The breeders in the Bieszczady Mountains also indicated a higher perception of risk for cattle than in the Knyszyńska Forest. In addition, breeders in the Białowieska Forest also indicated a higher perception of risk for humans than in the Knyszyńska Forest (Fig 6).

## Discussion

As expected, our study confirms a substantial risk of contacts between European bison and cattle in the study areas. Nevertheless, not all hypotheses were confirmed. These relationships appear reasonable when considering the management of the European bison population and cattle.

The hypothesis about the risk of contacts between European bison and cattle was confirmed at all sites, but surprisingly, similar risks were noted in the Knyszyńska and Borecka Forests. In the Knyszyńska Forest, European bison often live in open areas and cause significant damage to agricultural crops [11], while in the Borecka Forest these animals usually remain within a forest complex. However, it seems that this is due to the agricultural characteristics of these areas. The Podlaskie Voivodeship, where the Knyszyńska Forest is located, is characterized by the highest cattle population in Poland [60], but it varies locally. Although there is no detailed information about the cattle population in the studied communes, the local richness of the grazing area can be determined based on the area of pastures in the commune. The communes around the Knyszyńska Forest have a lower share of pasture land than those in the other sites, with pasture accounting for an average of 13% of the agricultural land, compared to about 25.7% around the Borecka Forest [61], 17.7% around the Białowieska Forest and 21.3% for the Bieszczady mountains [61]. It can hence be assumed that the European bison in the Knyszyńska Forest have a much lower chance of encountering cattle on pastures than in other study areas. Therefore, no significant difference in the risk of contact was found between the Knyszyńska Forest and the Borecka Forest.

Our results indicate that even in the areas where European bison live mainly in the forest complex, i.e. Borecka Forest, the risk of contacts between European bison and cattle may also occur. Moreover, the risk of pathogen transmission, resulting from contacts, seems to be higher in the Białowieska Forest and the Bieszczady Mountains. However, the risk of viral pathogen transmission seems to be higher in the case of the Białowieska Forest, due to more direct contacts, and parasitic pathogens are more likely in the Bieszczady Mountains, due to more frequent occurrence in the same pastures at different times. For example, in the case of protozoan parasites of the genus *Eimeria*, transmission occurs easily via sporulated oocysts contaminating pastures and water sources due to their direct life cycle [62]. In addition, some oocysts are able to survive the winter and become infectious at the beginning of the grazing season [48,63]. It is also worth mentioning that the Bieszczady Mountains can be distinguished from the other study areas by their land cover. In the Bieszczady Mountains, villages are located between large forest areas [55,56], which means that even if European bison roam in a forest complex, the relative distance to cattle may be small; however, the breeders did not report seeing European bison. This could indicate fewer estimated contacts between European bison and cattle in our study. For example, the spirurid nematodes *Thelazia gulos*a and *T*. *skrjabini* are transmitted between hosts (European bison and cattle) via *Musca* flies, which are vectors and intermediate hosts for *Thelazia* spp. [9]. According to Filip-Hutsch et al. [6], cattle may play a role in the transmission of *Thelazia* nematodes to free-living European bison. Individuals of genus *Musca* can fly over a distance of several kilometers [64].

Not surprisingly, our hypothesis indicating that the risk of contacts between European bison and cattle is dependent on the distance of cattle pastures from human settlements was confirmed. European bison avoid human infrastructure, including settlements, as shown in previous studies [65–67]. Human settlements are therefore an important factor in reintroduction plans for this species [68,69]. Even more surprising was that the chance of potential contacts throughout the year was confirmed, not just in spring and fall. Nevertheless, the greatest risk of contact was still associated with the spring months, when the cattle are on the pastures and European bison are still outside the forest complex, and the fall months, when cattle are still on the pastures and European bison are already outside the forest complex. The temporal intensity of the pathogen transmission pattern depends on the wildlife species and cattle management strategy [70–73]. Therefore, temporal change in risk should be considered in the field and in the context of wildlife behavior. However, it is important to evaluate wildlife management methods, because the highest risk of contacts between European bison and cattle was found in the Białowieska Forest and the Bieszczady Mountains, i.e., in the most numerous European bison populations in Poland [53]. This was also partly confirmed by the differences in risk perception for cattle (as well as humans), with the lower risk found in breeders from villages around the Borecka Forest.

Our findings suggest that it is possible to minimize the contacts between European bison and cattle by changing management practices. One important mechanism is to keep grazing areas as close as possible to settlements, which can significantly limit the possibility of direct or indirect contact between European bison and cattle. It may be possible to minimize the potential contacts by shortening cattle grazing time in pastures. It should be kept in mind that pathogen transmission between European bison and cattle is possible even in regions where European bison do not move beyond forest complexes and do not cause significant damage to agriculture. However, the risk seems to be greater when European bison populations are large and dispersal beyond forest complexes becomes the norm for these populations, especially when cattle are grazed in large numbers.

## Supporting information

S1 Table Main data obtained from the questionnaires. (DOCX)
