# Supplementary material for: Contact between European bison and cattle from the cattle breeders’ perspective, in the light of the risk of pathogen transmission
Source: PLoS One. 2023 May 3;18(5):e0285245. doi: 10.1371/journal.pone.0285245 (PMC10155960; doi:10.1371/journal.pone.0285245)
Supplement: S1 Table — (DOCX) [file pone.0285245.s001.docx]

**S1. Table.** **Main data obtained from the questionnaires**

|  | Białowieska F | Bieszczady Mts. | Borecka F. | Knyszyńska F. |
| --- | --- | --- | --- | --- |
| *Number of responses indicating given contact type* | | | | |
| NO CONTACT | 21 | 24 | 42 | 44 |
| SHARING PASTURES | 6 | 22 | 2 | 4 |
| AT DISTANCE | 7 | 4 | 6 | 3 |
| SEPARATED | 11 | 1 | 0 | 0 |
| NEAR | 9 | 0 | 1 | 1 |
| Total | 54 | 51 | 51 | 52 |
| *Distance of pasture to settlements (km)* | | | | |
| Mean | 0,76 | 0,42 | 0,49 | 0,25 |
| Min | 0 | 0,1 | 0 | 0 |
| Max | 4 | 2 | 4 | 3 |
| *Distance of pasture to forest (km)* | | | | |
| Mean | 1,48 | 0,27 | 1,37 | 1,96 |
| Min | 0 | 0 | 0 | 0 |
| Max | 8 | 2 | 10 | 10 |
| *Cattle - risk assessment form contacts with European bison. Responses to question: "Do you think direct contact with European bison poses a risk to cattle or humans?", answers: 1) Definitely not, 2) Rather not, 3) Hard to say, 4) Rather yes, 5) Definitely yes (transformed do Likert scale 1-5)* | | | | |
| Mean | 3,31 | 3,71 | 1,96 | 2,90 |
| Standard error | 0,18 | 0,14 | 0,14 | 0,15 |
| Median | 3 | 4 | 2 | 3 |
| *Human - risk assessment form contacts with European bison. Responses to question: "Do you think direct contact with European bison poses a risk to cattle or humans?", answers: 1) Definitely not, 2) Rather not, 3) Hard to say, 4) Rather yes, 5) Definitely yes (transformed do Likert scale 1-5)* | | | | |
| Mean | 3,90 | 3,22 | 1,96 | 3,16 |
| Standard error | 0,17 | 0,17 | 0,15 | 0,18 |
| Median | 4 | 3 | 2 | 3 |
|  |  |  |  |  |
|  |  |  |  |  |

S1.A. Table. Answers on contact type, distance of pasture to settlements and forest, risk to cattle and human assessment.

S1.B. Table. Number of declared presence of cattle on pastures and potential contact with European bison in given month

|  | JAN | FEB | MAR | APR | MAY | JUN | JUL | AUG | SEP | OCT | NOV | DEC |
| --- | --- | --- | --- | --- | --- | --- | --- | --- | --- | --- | --- | --- |
| Cattle | 12 | 12 | 18 | 30 | 198 | 206 | 206 | 206 | 204 | 180 | 77 | 18 |
| E. bison | 35 | 34 | 35 | 33 | 27 | 14 | 12 | 12 | 29 | 29 | 28 | 34 |
